# The Role of Nrf2 Transcription Factor and Sp1-Nrf2 Protein Complex in Glutamine Transporter SN1 Regulation in Mouse Cortical Astrocytes Exposed to Ammonia

**DOI:** 10.3390/ijms222011233

**Published:** 2021-10-18

**Authors:** Katarzyna Dąbrowska, Katarzyna Skowrońska, Mariusz Popek, Jan Albrecht, Magdalena Zielińska

**Affiliations:** Department of Neurotoxicology, Mossakowski Medical Research Institute, Polish Academy of Sciences, 5 Pawińskiego Str., 02-106 Warsaw, Poland; kdabrowska@imdik.pan.pl (K.D.); kskowronska@imdik.pan.pl (K.S.); mpopek@imdik.pan.pl (M.P.); jalbrecht@imdik.pan.pl (J.A.)

**Keywords:** astrocytes, glutamine, SN1 (SNAT3), Nrf2, Sp1, glutamine transport

## Abstract

Ammonia toxicity in the brain primarily affects astrocytes via a mechanism in which oxidative stress (OS), is coupled to the imbalance between glutamatergic and GABAergic transmission. Ammonia also downregulates the astrocytic N system transporter SN1 that controls glutamine supply from astrocytes to neurons for the replenishment of both neurotransmitters. Here, we tested the hypothesis that activation of Nrf2 is the process that links ammonia-induced OS formation in astrocytes to downregulation and inactivation of SN1 and that it may involve the formation of a complex between Nrf2 and Sp1. Treatment of cultured cortical mouse astrocytes with ammonia (5 mM NH_4_Cl for 24 h) evoked Nrf2 nuclear translocation, increased its activity in a p38 MAPK pathway-dependent manner, and enhanced Nrf2 binding to *Slc38a3* promoter. Nrf2 silencing increased SN1 mRNA and protein level without influencing astrocytic [^3^H]glutamine transport. Ammonia decreased SN1 expression in Nrf2 siRNA treated astrocytes and reduced [^3^H]glutamine uptake. In addition, while Nrf2 formed a complex with Sp1 in ammonia-treated astrocytes less efficiently than in control cells, treatment of astrocytes with hybrid-mode inactivated Sp1-Nrf2 complex (Nrf2 silencing + pharmacological inhibition of Sp1) did not affect SN1 protein level in ammonia-treated astrocytes. In summary, the results document that SN1 transporter dysregulation by ammonia in astrocytes involves activation of Nrf2 but does not require the formation of the Sp1-Nrf2 complex.

## 1. Introduction

Astrocytes maintain the metabolic, ionic, and neurotransmitter homeostasis in the brain [1]. Their key contribution to neurotransmission consists in supplying neurons with glutamine, a precursor of the amino acid neurotransmitters glutamate and γ-aminobutyric acid (GABA) [1,2] Glutamine is synthesized in astrocytes from ammonium ions (ammonia) and glutamate by glutamine synthetase, whereupon it leaves astrocytes and part of it is transferred to neurons; this chain of reactions is defined as a glutamine-glutamate-GABA cycle [3,4,5]. Glutamine out-transport from astrocytes is an active process, mainly mediated by the astroglia-specific glutamine transporter SN1. Under hyperammonemic conditions, glutamine synthesized in excess in astrocytes potentiates the direct deleterious effects of ammonia on astrocytic function, both by enhancing ammonia-induced oxidative/nitrosative stress (ONS) in the cells (the Trojan Horse hypothesis) and by increasing their osmotic burden [4,6]. Induction by ammonia of excessive glutamine accumulation is not only due to its increased synthesis but is also contributed by its impaired out-transport [7] from astrocytes, a dysfunction specifically related to decreased expression and activity of SN1 transporter function [8].

Reactive oxygen species (ROS) generated by ONS induce stress-inducible proteins, such as mitogen-activated protein kinase (MAPK) 1 and 2 [9,10], which results in the activation of nuclear Nrf2 transcription factor involved in the transcriptional regulation of cell defense genes of Phase I and Phase II drug-metabolizing enzymes [11]. In cell defense regulation driven by Nrf2 activation, glutamine metabolism is stimulated towards conversion to glutathione and tricarboxylic acid (TCA) cycle metabolites [12]. Nrf2 activation is mediated, among other mechanisms, by the PI3K-Akt or p38 MAPK signaling pathways, as was shown in proliferating cells [12] and rat astrocytes upon Mn^2+^ [13] or arachidonic acid [14] exposure. Ammonia-induced ROS formation and astrocytic swelling are mediated by a complex chain of reactions involving MAPK/ERK and PI3K/AKT signaling [15,16].

Here we tested the hypothesis that activation of Nrf2 is the process that links ammonia-induced OS to downregulation and inactivation of SN1. This idea was born from the following premises: (i) ammonia is a strong Nrf2 driver in astrocytes [17,18,19] and (ii) outside the realm of ammonia and the central nervous system, Nrf2 controls the expression of SN1 and, subsequently, glutamine delivery for glutathione synthesis in the acidotic kidney [20]. We also anticipated the possible role of the Sp1 transcription factor in this response. The anticipation was based on both experimental data documenting cooperation of Nrf2 with Sp1 in the regulation of transcription [21] (for recent references see [22]) and evidence that the Sp1 transcription factor binding to the *Slc38a3* promoter is involved in the regulation of transcription of SN1 [23,24]. Herein we also tested the assumption that functional interaction of Nrf2 with Sp1 may require the formation of a complex between the two proteins.

## 2. Results

### 2.1. Ammonia Increases Nrf2 Nuclear Protein Level and Its Activity in Mouse Cultured Astrocytes

**Figure 1:**
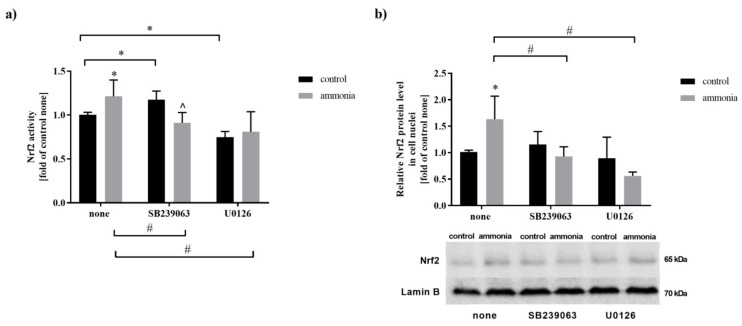
The effect of MAPK, and MEK1/2 inhibitors in the absence or presence of ammonia on the Nrf2 activity and Nrf2 nuclear protein level. (**a**) Nrf2 activity in mouse cortical astrocytes after exposure to ammonia, MAPK, and MEK1/2 inhibitors. Results are the mean ± SD (*n* = 4). (*) *p* < 0.05 vs. control none; (#) *p* < 0.05 vs. ammonia none; (^) *p* < 0.05 vs. control SB239063; two-way ANOVA, Bonferroni post hoc test. (**b**) Nrf2 protein level in cell nuclei of mouse cortical astrocytes after exposition to ammonia, MAPK, and MEK1/2 inhibitors. Results are the mean ± SD (*n* = 4). (*) *p* < 0.05 vs. control none; (#) *p* < 0.05 vs. ammonia none; (^) *p* < 0.05 vs. control SB239063; two-way ANOVA, Bonferroni post hoc test.

Nrf2 transcription factor activity was analyzed using a commercially available assay. In the presence of ammonia, Nrf2 activity increased by 21% in cultured mouse astrocytes (Figure 1a). In the absence of ammonia, Nrf2 activity increased by 17% after treatment with SB239063 (p38 MAPK inhibitor) and decreased by 26% after treatment with U0126 (MEK1/2 inhibitor) (Figure 1a). However, in the presence of ammonia, in cells exposed to SB239063, Nrf2 activity decreased by 22% and was not altered in cells exposed to U0126 compared to respective control (Figure 1a). As the active Nrf2 is present in the cell nuclei, the nuclear protein level was analyzed. Ammonia increased by 63% Nrf2 level in astrocytic nuclei compare to control while both SB239063 and U0126 decreased their level in ammonia-treated cells but not in untreated cells (Figure 1b).

### 2.2. Ammonia Enhances Nrf2 Binding to the Slc38a3 Promoter Region

**Figure 2:**
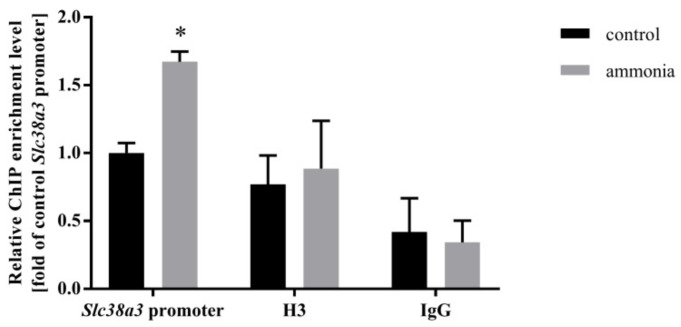
Nrf2 transcription factor binding to the *Slc38a3* promoter region in mouse astrocytes in the absence or presence of 5 mM ammonia for 24 h. Histone H3 was used as a positive control and IgG as a negative control. Results are the mean ± SD (*n* = 4). (*) *p* < 0.05 vs. control, two-way ANOVA, Bonferroni post hoc test.

Verification of the role of the Nrf2 transcription factor in the regulation of SN1 transporter in astrocytes was analyzed by Nrf2 binding to the *Slc38a3* promoter region in the ChIP experiments. An enrichment of Nrf2 binding to the *Slc38a3* promoter region was observed in both control and ammonia-treated astrocytes versus negative control but was more pronounced in ammonia-treated astrocytes (Figure 2).

### 2.3. Ammonia Decreases SN1 Expression in Astrocytes upon Nrf2 siRNA Transfection

**Figure 3:**
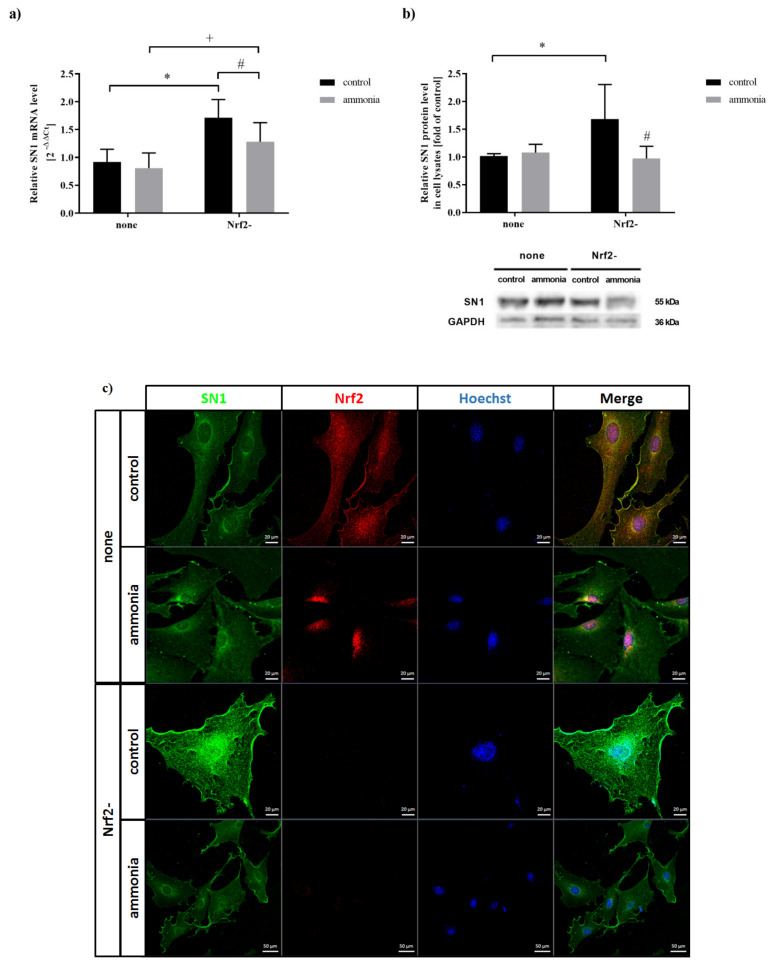
The effect of Nrf2 silencing (Nrf2-) on SN1 expression in mouse astrocytes in the absence or presence of 5 mM ammonia for 24 h. (**a**) SN1 mRNA level. (**b**) SN1 protein level. Results are the mean ± SD (*n* = 4). (*) *p* < 0.05 vs. control none; (#) *p* < 0.05 vs. control Nrf2-; (+) *p* < 0.05 vs. ammonia none; two-way ANOVA, Bonferroni post hoc test. (**c**) The expression of SN1 and Nrf2 in astrocytes before (none) and following Nrf2 silencing (Nrf2-). Image of SN1 (green, left), Nrf2 (red), nucleus (blue, Hoechst), and merged images (right).

The efficiency of Nrf2 silencing (Nrf2-) in mouse cortical astrocytes (62% in the absence, and by 56% in the presence of ammonia) was verified by Western Blot [(Supplementary Figure S1); for immunocytochemical demonstration see (Figure 3c)]. In the absence of ammonia, Nrf2 silencing upregulated mRNA (by 86%) (Figure 3a) and protein level (by 68%) of SN1 (Figure 3b,c). In cultures transfected with Nrf2 siRNA and treated with ammonia, the SN1 mRNA (Figure 3a, by 17%) and protein (Figure 3b, by 42% and 3c) level decreased as compared to Nrf2- control. The above results corroborate with confocal microscopy, demonstrating increased SN1 expression in Nrf2- control cells and documenting translocation of Nrf2 to the nuclei of astrocytes after ammonia treatment (Figure 3c).

### 2.4. Ammonia Decreases [^3^H]glutamine Uptake in Cultured Astrocytes upon Nrf2 siRNA Transfection

The effect of Nrf2 silencing (Nrf2-) on SN1 expression was functionally verified in the [^3^H]glutamine uptake experiments. Ammonia treatment decreased both, total (by 39%) and system N-mediated (by 50%) [^3^H]glutamine uptake in astrocytes treated with Nrf2 siRNA (Table 1). However, Nrf2 silencing itself did not alter [^3^H]glutamine uptake compared to cells in which Nrf2 was expressed (Table 1).

### 2.5. Nrf2 Silencing Decreases Expression of Nrf2 Target Genes in Mouse Cultured Astrocytes

**Figure 4:**
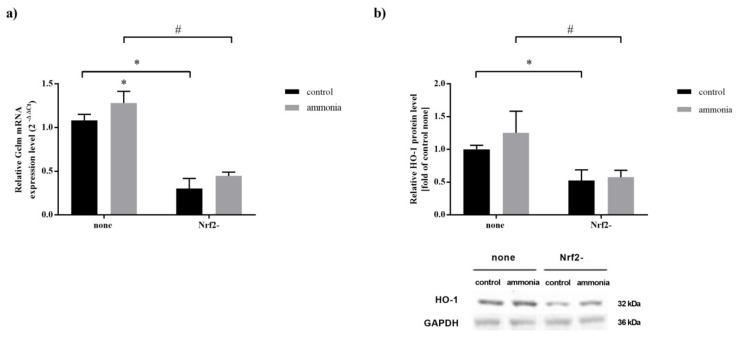
The effect of Nrf2 silencing (Nrf2-) on the Gclm mRNA level and HO-1 protein level in mouse astrocytes in the absence or presence of 5 mM ammonia for 24 h. (**a**) Gclm mRNA level. (**b**) HO-1 protein level. Results are the mean ± SD (*n* = 4). (*) *p* < 0.05 vs. control none; (#) *p* < 0.05 vs. ammonia none; two-way ANOVA, Bonferroni post hoc test.

The effect of ammonia and Nrf2 silencing (Nrf2-) on the Gclm mRNA level and HO-1 protein level in astrocytes was analyzed by qPCR and Western blot analysis. Ammonia increased (by ~20%) Gclm mRNA level (Figure 4a, none) in astrocytes in which Nrf2 was not silenced. However, Nrf2 silencing (Nrf2-) resulted in a decrease in Gclm mRNA level in control and ammonia-treated astrocytes (by 72% and 65%, respectively) (Figure 4a, Nrf2-) and HO-1 protein level (by 48% and 54%, respectively) (Figure 4b, Nrf2-).

### 2.6. Sp1-Nrf2 Complex Formation

**Figure 5:**
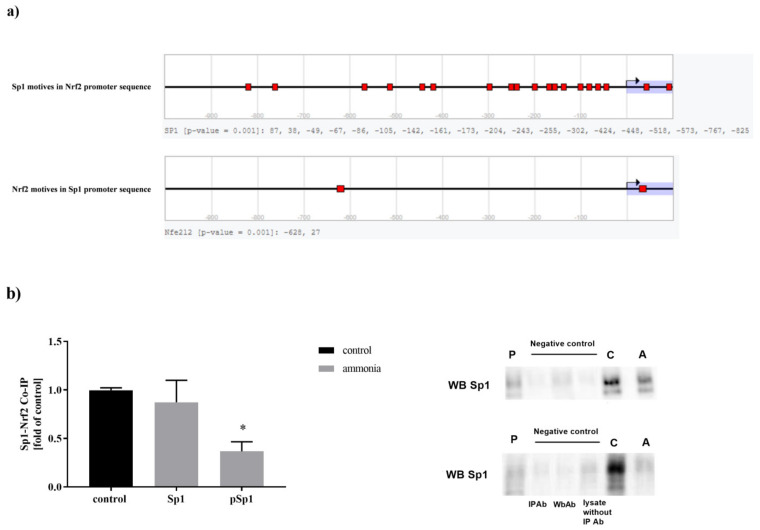
(**a**) In silico analysis of Sp1 and Nrf2 motives in Nrf2 and Sp1 promoter sequences respectively using the DBTSS database. (**b**) Co-immunoprecipitation of Sp1 and phosphorylated Sp1 (pSp1) with Nrf2 transcription factor in mouse cortical astrocytes in the absence or presence of 5 mM ammonia. The left panel shows summary data for mean densitometry of co-immunoprecipitation immunoblots, right panel, the representative co-immunoprecipitation immunoblot. Results are the mean ± SD (*n* = 4). (*) *p* < 0.05 vs. control; one-way ANOVA, Dunnett’s post hoc test.

Nrf2 and Sp1 form a protein complex that may regulate the expression of the target genes [20]. To confirm that Nrf2 and Sp1 protein complex is formed in mouse cortical astrocytes, we performed in silico analysis of Nrf2 and Sp1 transcription factor motives in the promoter sequence of other transcription factors using the TF Binding Site Search tool of DBTSS Database [kero.hgc.jp/tool/tf_search.html]. Both Nrf2 and Sp1 motives are present in the promoter sequences of Sp1 and Nrf2, respectively (Figure 5a). The in silico analysis results corroborate with co-immunoprecipitation experiments in vitro conducted on control and ammonia-treated astrocytes (Figure 5b). Moreover, the ability of Nrf2 to form complexes with phosphorylated Sp1 (pSp1) was significantly lower (decreased by 63%) in ammonia-exposed cells (Figure 5b).

### 2.7. Ammonia Does Not Affect SN1 Protein Level and [^3^H]glutamine Transport in Astrocytes with Inactivated Sp1-Nrf2 Complex

**Figure 6:**
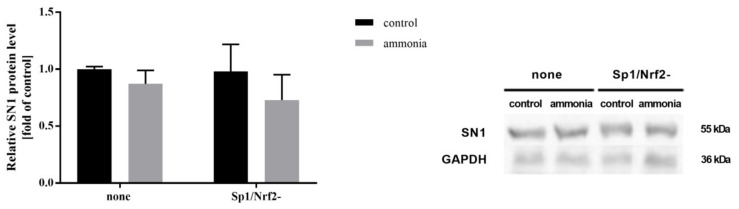
SN1 protein level in astrocytes with inactivated Sp1 and Nrf2 (Sp1/Nrf2-) complex in the absence or presence of 5 mM ammonia. The left panel shows summary for densitometric data, the right panel, a representative immunoblot. Results are the mean ± SD (*n* = 4).

Next, we analyzed the effect of Sp1-Nrf2 complex inactivation (Sp1/Nrf2-) on SN1 protein level as well as on the total, and system N-mediated [^3^H]glutamine transport in astrocytes exposed to ammonia. None of the changes were indicated in SN1 protein level (Figure 6) and [^3^H]glutamine transport (Supplementary Table S1) after ammonia treatment.

## 3. Discussion

We demonstrated that exposure of cortical astrocytes to ammonia results in p38 MAPK-regulated activation of Nrf2 transcription factor and that ammonia-induced Nrf2 nuclear translocation affects the expression but not the function of the SN1 glutamine transporter.

Ammonia-induced ROS generation results in nitration of proteins, oxidation of RNA, and alteration of gene expression and cellular signaling [25,26]. In addition, ammonia detoxifying end-product glutamine modulates the osmotic and redox status of the cell, and may also act as a positive or negative modulator of transcription factors activation [27,28]. Upon stressful conditions, the transcription factor Nrf2 binds to the ARE elements of the promoter regions of the cell defense genes, such as NAD(P)H quinone dehydrogenase (NQO1), heme oxygenase 1 (HO-1), and the two subunits of glutamate-cysteine ligase (Gcl): catalytic (Gclc) and regulatory (Gclm), the latter rate-limiting for glutathione synthesis [29,30]. Our results showed that in astrocytes after ammonia exposure Nrf2 translocates to the nuclei, and Nrf2 nuclear protein is increased. One of the signaling pathways involved in Nrf2 activation is the PI3K-Akt pathway, which is highly active in proliferating cells [12]. The mechanism involving PI3K-Akt-mediated activation has been shown to operate in rat astrocytes upon Mn^2+^ exposure [13,31] and in mouse astrocytes treated with ammonia [15]. The other mechanism regulating Nrf2 activity involves the MAPK cascade. In astrocytes exposed to excess glutamine, intracellular accumulation of glutamine causes osmotic stress and activates the MAPK/p38 cascade [28]. In ammonia-treated astrocytes, in the present study, inhibition of the p38 MAPK pathway reduced, whereas inhibition of mitogen-activated protein kinase kinases 1/2 (MEK1/2) did not alter Nrf2 activity. However, in control cells, inhibition of p38 MAPK increased the Nrf2 activity. The results suggest that ammonia exposure favors the Nrf2 activation mode toward being p38 MAPK dependent. Interestingly, in carbon monoxide-treated astrocytes, p38 pathway upregulation likewise led to the activation of Nrf2 [16].

Recently, it was shown that Nrf2 regulates the SN1 transporter expression in mouse kidneys during metabolic acidosis [20]. In the above study, Nrf2 depletion decreased SN1 mRNA and protein level in physiological conditions, and fully abrogated expression of SN1 during metabolic acidosis. In contrast, in our study, Nrf2 depletion decreased SN1 mRNA and protein levels in ammonia-treated astrocytes but upregulated SN1 transporter expression in control astrocytes. Collectively, the results support the view that in mouse cortical astrocytes, SN1 expression depends on the activation of the Nrf2 transcription factor. Indeed, ChIP analyses showed enriched Nrf2 recruitment over the *Slc38a3* promoter region in ammonia-treated astrocytes. The association of histone H3 with the *Slc38a3* promoter was unchanged, indicating the relationship to the reduced histone H3 level by ammonia [32] and its involvement in the SN1 transcription activation [23]. The literature data and unaltered SN1 expression in astrocytes upon ammonia exposure collectively confirm the interaction of SN1 with histone H3.

Next, we tested whether SN1 expression changes observed in ammonia-treated astrocytes with depleted Nrf2 affect glutamine transport, as previously characterized for Sp1 [24,31,33]. Here, ammonia decreased total and system N-mediated [^3^H]glutamine uptake to astrocytes with depleted Nrf2. However, [^3^H]glutamine uptake was unaltered in ammonia-treated astrocytes non-transfected with Nrf2 siRNA. Of note, the preponderance of SN1 transporter to promote [^3^H]glutamine uptake or release very much depends upon subtle changes in ambient conditions [34].

As mentioned earlier, Nrf2 activation by OS triggers cell defense genes expression, and the response includes enzymes involved in glutathione synthesis [12]. It has been repeatedly documented that ammonia induces astrocytic glutathione accumulation in vitro [35,36] and its extracellular increase in the prefrontal cortex of rats after brain ammonia infusion [37]. SN1 transporter, which is responsible for the export of glutamine from the astrocytes [7,24,33], might be involved in cell defense. This possibility was suggested in the study by Lister et al. conducted in mouse kidney depleted of Nrf2, in which significantly altered SN1 mRNA level was observed [20]. The Nrf2-dependent Gclm mRNA increase in ammonia-treated astrocytes is in line with previously documented glutathione changes in ammonia-affected astrocytes [35]. The results suggest that glutamine transported by SN1 might contribute to glutathione synthesis in ammonia-treated astrocytes. Indeed, SN1 silencing in astrocytes resulted in the intracellular increase in glutathione and a tendency towards a decrease in glutamine content in control astrocytes [7]. Lister and colleagues found liver and brain tissues to be insensitive to SN1 induction upon metabolic acidosis, since in their hands Nrf2 did not control basal SN1 mRNA expression level in there [20]. However, the absence of changes in total cell homogenates observed in the latter study cannot rule out the possibility of cell-specific Nrf2-dependent control of SN1 in neonatal astrocytes as documented in our study. Moreover, it should be emphasized that metabolic acidosis did not change Nrf2 mRNA level in the kidney and Nrf2-dependent glutathione level remained almost unmeasurable during metabolic acidosis [20]. Inversely, cortical astrocytes depleted with Nrf2 upon ammonia exposure presented a decrease in Gclm mRNA level and a decrease in the HO-1 protein level (Figure 6).

In our study, the Sp1-Nrf2 protein complex was detected in mouse cortical astrocytes treated with ammonia. Recently, we documented the decrease in SN1 mRNA level after PKC activation in ammonia-exposed astrocytes, which purportedly could be related to a decreased level of the Sp1 phosphorylated form (pSp1) [24,33] albeit this observation cannot be interpreted as reflecting ammonia-induced Sp1 phosphorylation. In this study, ammonia treatment significantly decreased the protein level of pSp1 in astrocytes, corroborating with our previous observation [24]. Interestingly, neither alterations of SN1 protein level nor of [^3^H]glutamine transport have occurred upon inactivation of the Sp1-Nrf2 complex. It is likely that additional components of such a complex (e.g., c-Jun; [16]), might be an as yet unraveled mechanism contributing to Nrf2-mediated increases in SN1 protein level and [^3^H]glutamine transport in astrocytes. Therefore, the roles of the individual components in the regulation of the SN1 transporter in astrocytes deserve further investigation. Another explanation may include the opposing effects of Nrf2 and Sp1 in the SN1 regulation control in mouse astrocytes. It was previously suggested that in mouse cortical astrocytes the Sp1 transcription factor is involved in the SN1 regulation as the possible silencer of SN1 expression [24].

While the results of this study do not resolve whether Sp1 is required for the mediation of this effect by Nrf2, it indicates that in contrast to SN1 expression, the formation of the Sp1-Nrf2 complex is not required here. Of note, other reports do not define the role of Sp1 as a mediator of Nrf2-dependent increase in glutathione synthesis in astrocytes by other toxic stimuli [38,39].

## 4. Materials and Methods

### 4.1. Materials

Cell culture plates and medium were obtained from Sigma-Aldrich (St. Louis, MO, USA), fetal bovine serum (FBS) from Biosera (Nuaillé, France), and antibiotic antimycotic from Gibco (Thermo Fisher Scientific, Waltham, MA, USA). All chemicals used in this study were commercially available and of the purest grade.

### 4.2. Cell Culture and Treatment

Primary cultures of astrocytes were prepared from cortices of 7-day-old C57BL6/J mice from the animal colony of the Mossakowski Medical Research Institute, Polish Academy of Sciences, in Warsaw according to the earlier described protocol [40,41]. Briefly, the isolated cortex was passed through 80 μM and then 40 μM nylon netting filters into Dulbecco’s Modified Eagle’s Medium (DMEM) containing 20% FBS. The medium was changed twice a week, gradually reducing the content of FBS, reaching 10% in the third week of cell culture. Dibutyryl cyclic AMP (Sigma-Aldrich, St. Louis, MO, USA) was added to the culture medium in the third week of culturing to promote morphological differentiation of the cells. The cells were cultured at 37 °C in an atmosphere of 95% O_2_ and 5% CO_2_. The standard protocol used in our laboratory includes checking cultured astrocyte purity immunocytochemically (Glial fibrillary acidic protein staining).

After 3 weeks of cell culture, astrocytes were exposed to 5 mM ammonium chloride (“ammonia”; Sigma-Aldrich, St. Louis, MO, USA) for 24 h. In the experiments analyzing the regulation of Nrf2 expression and activity by p38 MAPK and MEK1/2 kinases, cells were treated with 10 ÌM SB239063 (p38 MAPK inhibitor) or 10 ÌM U0126 (MEK1/2 inhibitor) (Sigma-Aldrich, St. Louis, MO, USA) for 24 h.

### 4.3. Nrf2 Transcription Factor Silencing

To down-regulate the expression of the Nrf2 transcription factor, astrocytes were transfected with the mix of four types of siRNA duplexes, each of which consisted of 21 nucleotides and was targeted for the different gene regions to obtain the most effective silencing. Sense strands were: 5′-AACTCTCAAGTTAATTTCTTA-3′, 5′-CACGCTGAAGGCACAATGGAA-3′, 5′-CCCGTGAGTCCTGGTCATCAA-3′, 5′-TAGCAATAATATGAAACTTTA-3′.

Cells were washed with phosphate-buffered saline (PBS, Sigma-Aldrich, St. Louis, MO, USA), trypsinized to detach cells from the plates, and plated at the density of 0.6 × 10^5^ cells per well in 24-well plates in 0.5 mL of DMEM with 10% FBS or at a density of 1.8 × 10^5^ cells per well in 6-well plates in 1.5 mL of DMEM with 10% FBS. Subsequently, astrocytes were transfected according to the fast-forward protocol provided by the manufacturer by the drop-wise addition of the transfection mixture consisting of siRNA duplexes, HiPerfect Transfection Reagent (Qiagen, Hilden, Germany), and OptiMEM (Gibco, Thermo Fisher Scientific, Waltham, MA, USA). Before the transfection, the mixture was incubated for 30 min at room temperature (RT) to form the complexes between transfection reagent and siRNA sequences. The final concentration of siRNA in each well was 5 nM and the transfection of the cells lasted 24 h in normal growth conditions.

### 4.4. Inactivation of the Sp1-Nrf2 Protein Complex

The Sp1-Nrf2 protein complex was down-regulated by silencing of the Nrf2 expression, as described above, while the Sp1 protein was decreased by pharmacological inhibition of astrocytes with 10 µM mithramycin A (Sp1 inhibitor, Sigma-Aldrich, St. Louis, MO, USA) for 24 h.

### 4.5. RNA Isolation and Real-Time qPCR Analysis

Total RNA was isolated from astrocytes using TRI Reagent (Sigma-Aldrich, St. Louis, MO, USA) and 1 μg was then reverse transcribed using the High-Capacity cDNA Reverse Transcriptase Kit (Applied Biosystems, Warrington, UK). The real-time PCR reactions containing 5 μL TaqMan Fast Universal PCR Master Mix (Applied Biosystems, Warrington, UK), 0.5 μL probe, and 1.5 μL of cDNA were run on 96-well plates using The Applied Biosystems 7500 Fast Real-Time PCR System. Experiments were performed according to a fast protocol: 20 s at 95 °C followed by 45 cycles of 3 s at 95 °C and 30 s at 60 °C. Taqman probes for SN1, Gclm, and endogenous control β-actin (Mm0120670_m1, Mm01324400_m1, and Mm00607939_s1, respectively) were provided by Applied Biosystems (Warrington, UK). The results were calculated and expressed according to the equation 2^−ΔΔCt^ giving the amount of the target, normalized to the endogenous control and Ct was a threshold cycle for target amplification [42].

### 4.6. Nuclear Protein Extraction

The extraction of nuclear proteins was performed using Nuclear Extraction Kit (#ab113477, Abcam, Cambridge, UK) according to the manufacturer’s protocol. Shortly, the astrocytes were prepared by incubation on ice for 10 min with pre-extraction buffer and centrifugation for 1 min at 12,000× *g*. The pellet was re-suspended in extraction buffer supplemented with DTT solution and protease inhibitor cocktail, incubated on ice for 15 min, sonicated, and centrifuged for 10 min at 14,000× *g*. Then, the supernatant was transferred to another tube and the protein concentration was measured using the Pierce BCA Protein Assay Kit (Thermo Fisher Scientific, Waltham, MA, USA).

### 4.7. Protein Isolation and Western Blot

Three-week-old astrocytes were scraped off from the plates and homogenized in RIPA buffer (0.01 M sodium phosphate (pH 7.2), 0.15 M sodium chloride, 0.1% sodium dodecyl sulfate, 1% sodium deoxycholate, 1% NP-40, 2 mM EDTA) containing protease and phosphatase inhibitor cocktails (1:100 and 1:200 respectively, Sigma-Aldrich, St. Louis, MO, USA) and sodium fluoride (50 mM, Sigma-Aldrich, St. Louis, MO, USA). The protein concentration was measured using the Pierce BCA Protein Assay Kit (Thermo Fisher Scientific, Waltham, MA, USA). Equal amounts of protein (30 μg) were subjected to Western blot analysis. The protein samples were denatured by boiling in SDS-PAGE loading buffer for 10 min at 95 °C, separated on an SDS polyacrylamide gel electrophoresis, and then transferred onto a nitrocellulose membrane. Prepared membranes were blocked and incubated overnight at 4 °C with primary antibodies against SN1 (1:800, #14315-1-AP, Proteintech, Manchester, UK), Nrf2 (1:800; #PA5-27882, Thermo Fisher Scientific, Waltham, MA, USA), phosphorylated Sp1 (1:500, #ab59257, Abcam, Cambridge, UK), lamin B1 (1:5000, #PA5-19468, Thermo Fisher Scientific, Waltham, MA, USA) followed by a 1 h incubation with HRP-conjugated anti-rabbit IgG (1:3000 for SN1, 1:4500 for Nrf2 and 1:8000 for lamin B) to detect those proteins by Clarity Western ECL Substrate (Bio-Rad Laboratories, Hercules, CA, USA). Then, after stripping with 0.1 M glycine (pH 2.9, Sigma-Aldrich, St. Louis, MO, USA) and 1 h blocking, the blots were incubated for 1 h at RT with an HRP-conjugated antibody against glyceraldehyde-3-phosphate dehydrogenase (GAPDH; 1:7500, Proteintech, Manchester, UK) and detected as described above. The chemiluminescent signal acquisition and densitometry analysis were performed using the G-Box System (SynGene, Cambridge, UK) and GeneTools Software (SynGene, Cambridge, UK).

### 4.8. Nrf2 Activity Assay

Nrf2 activity was analyzed using the Nrf2 Transcription Factor Assay Kit (#ab207223, Abcam, Cambridge, UK) according to the manufacturer’s protocol. Briefly, nuclear extracts were added to the plates pre-coated with an oligonucleotide containing Nr2 consensus binding site and incubated for 1 h at RT. Then, the primary antibody was added to the wells and the plate was incubated for 1 h at RT. After washing, HRP-conjugated secondary antibody was added to the wells and the samples were incubated for 1 h at RT. Subsequently, each well was washed and a developing solution was added. When the solution color turned from medium to dark blue, a stop solution was added and the absorbance was measured at 450 nm.

### 4.9. [^3^H]glutamine Uptake

Astrocytes were washed twice with Krebs buffer (29.5 mM NaCl, 1.13 mM KCl, 0.3 mM KH_2_PO_4_, 0.3 mM MgSO_4_, 11 mM glucose, 25 mM NaHCO_3_, 2.5 mM CaCl_2_) and pre-incubated in this buffer for 15 min at 37 °C. Next, the cells were incubated in the mixture of Krebs buffer supplemented with 0.1 μCi/mL L-[3,4-^3^H(N)-]glutamine (PerkinElmer, Waltham, MA, USA; specific radioactivity 37 MBq/mL) and 0.1 mM unlabeled glutamine. To block systems other than system N in the experiments analyzing system N-mediated [^3^H]glutamine uptake, the mixture also contained 10 mM L-Ala and 10 mM L-Leu (Sigma-Aldrich, St. Louis, MO, USA) [9]. The incubation was terminated after 4 min by a triple wash of the cells using cold Krebs buffer. The astrocytes were lysed by incubation with 0.5 mL of 1 N NaOH and the radioactivity of the cells was measured in a Wallac 1409 Liquid Scintillation Counter (Perkin-Elmer, Turku, Finland).

### 4.10. Chromatin Immunoprecipitation (ChIP)

The chromatin immunoprecipitation experiments were performed as described earlier [24]. In brief, 10^7^ astrocytes were cross-linked in 1% formaldehyde (Sigma-Aldrich, St. Louis, MO, USA). The formaldehyde quenching was performed by the addition of glycine in a final concentration of 0.125 M. Then, the cells were sonicated and the chromatin was immunoprecipitated overnight at 4 °C with Nrf2 antibody (1 μg, #PA5-27882, Thermo Fisher Scientific, Waltham, MA, USA), histone H3 (a positive control, #4620, Cell Signaling, Leiden, The Netherlands) or IgG (negative control, #2729, Cell Signaling, Leiden, The Netherlands). Part of each sample was taken as input before the incubation with specific antibodies. Subsequently, the samples were washed and de-crosslinked by dissolving them in the elution buffer supplemented with 5 M NaCl overnight at 65 °C. DNA from each sample was purified using a phenol:chloroform: isoamyl alcohol solution (#P3803, Sigma-Aldrich, St. Louis, MO, USA) with the addition of glycogen (Roche, Mannheim, Germany). The quantitative analysis of the experiments was performed in real-time qPCR reactions using Platinum Taq DNA Polymerase Kit (Invitrogen, Carlsbad, CA, USA), 10 mM dNTPs (Invitrogen, Carlsbad, CA, USA), and SYBR Green (solution 1:2000; Invitrogen, Eugene, OR, USA). The enrichment at the *Slc38a3* promoter region was normalized versus input and non-binding region. The primers used in this study were: *Slc38a3* promoter region (sense strand: 5′-AAACACTTGGAGGGGCTTCT-3′, antisense strand: 5′-CCTCGAAATCGGTGAAGTGT-3′), non-binding region (sense strand: 5′-CTCCTTGTACGGGTTGTT-3′, antisense strand: 5′-AATGATGTGCACAGCTGAA-3′) (Laboratory of DNA Sequencing and Oligonucleotide Synthesis IBB, Warsaw, Poland).

### 4.11. Immunocytochemistry

Astrocytes were plated on poly-L-lysine coated glass coverslips in 24-well plates and cultured for 4 days. After a triple wash with PBS, the cells were fixed with 4% paraformaldehyde for 20 min at RT. Subsequently, they were permeabilized with 0.25% Triton X-100/PBS for 30 min at RT and blocked in 10% normal goat serum/PBS for 1 h. Incubation with the primary antibody against Nrf2 (1:200, #66504-1-Ig, Proteintech, Manchester, UK) and SN1 (1:200, #14315-1-AP, Proteintech, Manchester, UK) was performed overnight at 4 °C and followed by a 1 h incubation with secondary goat anti-mouse IgG2b Alexa Fluor 546 antibody (1:500, Invitrogen, Waltham, MA, USA) and goat anti-rabbit IgG Alexa Fluor 488 antibody (1:1000, Invitrogen, Waltham, MA, USA) at RT in the dark. Afterward, to label cell nuclei the cells were washed with PBS and incubated for 20 min with Hoechst 33258 (H1398, Thermo Fisher Scientific, Waltham, MA, USA) stain solution (0.5 μg/mL) at RT in the dark. The cells were placed on the microscope slides using Dako Fluorescence Mounting Medium (Agilent Technologies, Carpinteria, CA, USA). Detailed pictures of the labeled astrocytes were taken using a confocal laser scanning microscope LSM 780 (Zeiss, Jena, Germany). An argon laser (488 nm) was used for the excitation of Alexa Fluor 488 and a diode 405 nm for the Hoechst staining. The images were processed using the ZEN 2012 (Zeiss, Jena, Germany). The studies were performed in the Laboratory of Advanced Microscopy Techniques, Mossakowski Medical Research Institute, Polish Academy of Sciences.

### 4.12. Co-Immunoprecipitation

The experiments were carried out according to the earlier described protocol with modifications [33,43]. In brief, astrocytes were lysed in RIPA buffer containing protease and phosphatase inhibitors and 50 mM sodium fluoride. One milligram of protein was pre-cleared by shaking with Pansorbin cells (Callbiochem, San Diego, CA, USA) at 4 °C for 1 h, and then collected supernatant was incubated with SN1 antibody (#14315-1-AP, Proteintech, Manchester, UK) overnight at 4 °C. Subsequently, proteins were precipitated for 2 h at 4 °C with protein-G agarose beads (Sigma-Aldrich, St. Louis, MO, USA), and the immune complexes were washed with RIPA buffer and with wash buffer (100 mM NaCl, 50 mM Tris-HCl, pH 7.5, 0.5% Nonidet P-40). The complexes were solubilized in SDS-PAGE loading buffer at 95 °C for 5 min and analyzed in Western blot experiments. Whole-cell lysates were used as a positive control and the following samples were used as negative controls: beads with the antibody used for immunoprecipitation (IP Ab, Sp1, pSp1), beads with the antibody used in Western blot analysis (IP Wb, Nrf2), and lysate without antibody used for immunoprecipitation (Sp1, pSp1).

### 4.13. Statistical Analysis

Statistical significance was checked by the one- or two-Way ANOVA test followed by Dunnett’s or Bonferroni post hoc test using GraphPad Prism 7.0 software (GraphPad Software, La Jolla, CA, USA). The normality of the data distribution was confirmed by the Kołmogorow–Smirnow test. A probability value of 0.05 or less was considered statistically significant.

## 5. Conclusions

In conclusion, the results document that SN1 transporter dysregulation by ammonia in cultured mouse astrocytes involves activation of Nrf2 but does not require the formation of the Sp1-Nrf2 complex. This research provides evidence that pathologically elevated ammonia activates the Nrf2 transcription factor in a p38 MAPK pathway-dependent manner and enhances Nrf2 binding to the *Slc38a3* promoter region, in this way playing a role in glutamine transport alterations in astrocytes. However, the relevance of these findings for understanding the role of Nrf2 in the regulation of SN1 transporter and SN1-mediated glutamine transport in the brain during hyperammonemia in vivo remains to be documented using more native systems.

## Figures and Tables

**Table 1:** The effect of Nrf2 silencing (Nrf2-) on total and system N-mediated [^3^H]glutamine uptake in astrocytes in the absence or presence of 5 mM ammonia for 24 h. Basal [^3^H]glutamine uptake was 42.93 ± 16.09 nmol/mg of protein x min.

	[^3^H]glutamine Uptake [Fold of Control Total None]	
	None	Nrf2-
Total		
Control	1.00 ± 0.17	1.01 ± 0.22
Ammonia	0.82 ± 0.15	0.62 ± 0.19 ^+^
		
System N		
Control	0.29 ± 0.14 *	0.24 ± 0.06 ^+^
Ammonia	0.21 ± 0.10 ^#^	0.12 ± 0.06 ^=,^^

Results are the mean ± SD (*n* = 4). (*) *p* < 0.05 vs. control total none; (^#^) *p* < 0.05 vs. ammonia total none; (^+^) *p* < 0.05 vs. control total Nrf2-; (^=^) *p* < 0.05 vs. ammonia total Nrf2-; (^^^) *p* < 0.05 vs. control (system *n*) Nrf2-; two-way ANOVA, Bonferroni post hoc test.

## Data Availability

Data available on request.

## Supplementary Materials

The following are available online at https://www.mdpi.com/article/10.3390/ijms222011233/s1.

## Abbreviations

ARE	Anti-oxidative response elements
ChIP	Chromatin immunoprecipitation
CNS	Central nervous system
DMEM	Dulbecco’s Modified Eagle’s Medium
FBS	Fetal bovine serum
GABA	Gamma-aminobutyric acid
Gcl	Glutamate-cysteine ligase
GAPDH	Glyceraldehyde 3-phosphate dehydrogenase
Gclc	Glutamate-cysteine ligase catalytic subunit
Gclm	Glutamate-cysteine ligase regulatory subunit
HO-1	Heme-oxygenase 1
Keap1	Kelch like-ECH-associated protein 1
MAPK	Mitogen-activated protein kinase
MEK 1/2	Mitogen-activated protein kinase kinase
NQO1	NAD(P)H quinone dehydrogenase
Nrf2	Nuclear factor erythroid-related 2-like 2
ONS	Oxidative/nitrosative stress
OS	Oxidative stress
PBS	Phosphate-buffered saline
pSp1	Phosphorylated specificity protein 1
RONS	Reactive oxygen-nitrogen species
ROS	Reactive oxygen species
RT	Room temperature
SN1	SNAT3, solute carrier family 38 member 3
Sp1	Specificity protein 1
TCA	Tricarboxylic acid
WT	Wild-type
