# Supplementary material for: The Role of Nrf2 Transcription Factor and Sp1-Nrf2 Protein Complex in Glutamine Transporter SN1 Regulation in Mouse Cortical Astrocytes Exposed to Ammonia
Source: Int J Mol Sci. 2021 Oct 18;22(20):11233. doi: 10.3390/ijms222011233 (PMC8538223; doi:10.3390/ijms222011233)
Supplement: Supplementary file 1 [file ijms-22-11233-s001.zip › Supplementary Figure 1.pdf]

Supplementary Figure S1

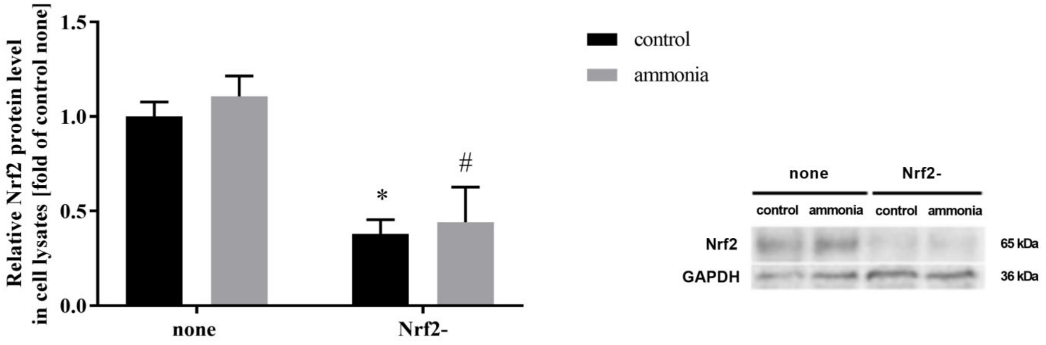

Figure S1. The efficiency of Nrf2 silencing in mouse cortical astrocytes. The left panel shows results of densitometry analysis, right the representative immunoblots. Results are mean±SD (n=4). (*)$p<0.05$ vs control none; (#)$p<0.05$ vs ammonia none; Two-Way ANOVA; Bonferroni posthoc test.
