# Supplementary material for: The Role of Nrf2 Transcription Factor and Sp1-Nrf2 Protein Complex in Glutamine Transporter SN1 Regulation in Mouse Cortical Astrocytes Exposed to Ammonia
Source: Int J Mol Sci. 2021 Oct 18;22(20):11233. doi: 10.3390/ijms222011233 (PMC8538223; doi:10.3390/ijms222011233)
Supplement: Supplementary file 1 [file ijms-22-11233-s001.zip › Supplementary Table 1.pdf]

Supplementary Table S1

Table S1. Total and system N-mediated [^{3}H]glutamine transport in astrocytes with silenced Sp1-Nrf2 (Sp1/Nrf2-) complex in the absence or presence of 5mM ammonia. Basal [^{3}H]glutamine uptake was 42.93±16.09 nmol/mg of protein x min.

	**[^{3}H]glutamine Uptake [Fold of Control Total None]**		**[^{3}H]glutamine Efflux [Fold of Control Total None]**	
	**None**	**Sp1/Nrf2-**	**None**	**Sp1/Nrf2-**
Total				
control	1.00±0.17	1.12±0.08	1.03±0.08	1.18±0.07
ammonia	0.82±0.15	0.94±0.20	0.98±0.13	0.91±0.26^{+}
System N				
Control	0.29±0.14*	0.24±0.07^{+}	1.23±0.40	1.36±0.17
ammonia	0.21±0.10$^{\#}$	0.24±0.05$^{=}$	1.07±0.10	1.20±0.05$^{=}$

Results are mean±SD (n=4). (*)p<0.05 vs control total none; (#)p<0.05 vs ammonia total none; (+)p<0.05 vs control (total) Sp1/Nrf2-; (=)p<0.05 vs ammonia (total) Sp1/Nrf2-; Two-Way ANOVA, Bonferroni posthoc test.
